# Entrepreneurship education at universities: challenges and future perspectives on online game implementation

**DOI:** 10.1007/s41959-020-00043-3

**Published:** 2021-02-02

**Authors:** Takuji Takemoto, Hiroko Oe

**Affiliations:** 1 Headquarters for Regional Revitalization, University of Fukui, Fukui, Japan; 2 The Business School, Bournemouth University, Poole, UK

**Keywords:** Online game, Entrepreneurship, Higher education organisation, Beyond COVID-19 pandemic, Action learning circuit with games, Edtech

## Abstract

The study explored the challenges and potential of online gamification to develop actionable recommendations for entrepreneurship pedagogy in the phase of ‘new normal’. This study applied an experimental game which the authors developed, and a mixed method was applied to the data sets collected from the students: an open-ended survey of 91 students and in-depth interviews with 23 students. It has been found that the students perceiving activities with gamifications are good learning stimuli in entrepreneurial classrooms as a first step; then, they found it effective to learn and deepen their understanding of theories and models as a second step after the gaming activities, which is a reverse approach from a traditional business education approach. Gamification enables students to think critically on game scenarios via participation in gamifications, which can be strengthened and embedded in their mind by theoretical learning which follows the gaming activities. The findings of the study provide a practical guidance for entrepreneurship pedagogists with ‘activities first’ which will be followed by theoretical learning.

## Introduction

In business, companies, customers, and competition (the so-called 3Cs model) do not necessarily act rationally and their behaviour cannot be predicted. Therefore, it could be concluded that learning from lectures only is not an appropriate method of entrepreneurship and business education because it is sometimes not providing a real business scenario. The conventional passive approach to education cannot necessarily give students the real business dynamism. On this point, online gamification has been attracting pedagogists and researchers with a potential to enhance students’ learning performance in the complex systemic context with nonlinear relationships among business stakeholders.

However, a framework how to implement online gaming in entrepreneurship and business education has not yet been agreed on and developing specific guidelines with key points for curriculum designing has been on the agenda.

## Theoretical discussion

### Entrepreneurial education

Li and Wu (2019) suggested that entrepreneurial education should be aligned with team collaboration and interactions based on real business contexts within an organisation. Moreover, the core consideration when designing the entrepreneurial unit should be the promotion of self-efficacy and confidence (Nowiński et al. 2019). In the context of contemporary higher educational perspectives, it is important to bring students with different values and backgrounds together in the international classroom and to engage them with cross-cultural entrepreneurial modules (Stefanic et al. 2020).

It is inevitable for the pedagogists to consider the challenges the 4th Industrial Age have brought about to entrepreneurship education as it has created a fundamental change to entrepreneurial development (Zhang 2018). The relationship with technology has significant impacts on various aspects of business behaviour, which implies, even with or without COVID-19 influence, entrepreneurial education needs to catch up with the speedy transition of business scenarios to deliver effective learning modules to support developing entrepreneurial students with entrepreneurial mindset (e.g. Maaravi et al. 2020; Zhang 2018).

## Interactive learning based on a virtual setting

Gamification has attracted the attention of pedagogical researchers and practitioners due to its potential to enhance learning ability. The recent increased focus on ‘Edtech’ (educational technology) has highlighted the implications and potential of interactive online games to improve learners’ financial capabilities.

Deb and Bhatt (2020) emphasised the benefits of integrated digital learning to enhancing skill-oriented entrepreneurial education. They further noted that, in this context, employing business gamification in business units could potentially contribute to student learning. A virtual setting enables learners to experience a variety of different settings that cannot be achieved in real physical cases. Blended learning, which combines both online and offline learning perspectives, is one of the best approaches in this context (Stefanic et al. 2020).

In line with the positive impact and the continuing development of potential advanced technologies, Niebuhr and Tegtmeier (2019) suggested the use of specific assistive technologies, such as virtual reality (VR), as digital learning tools in order to involve entrepreneurship factors in the learning process. Similarly, Goldstein and Gafni (2019) and Srivastava et al. (2019) suggested the teaching of entrepreneurship through virtual learning settings based on multicultural teamwork. This enhances collaborative and independent learning by boosting student motivation to understand the business context in a more critical manner.

### Gamification and entrepreneurship education

Learning based on digitalised learning measures, including Edtech, is attracting increasing attention from researchers and educators. The positive impact of gamification, particularly in the context of entrepreneurial education, has been discussed in pedagogical studies (Antonaci et al. 2015).

This approach has been a topic of discussion for several years, with practitioners who studied entrepreneurial units at university providing some testimonials to suggest that gamification had a positive impact on students’ subsequent business behaviour and practices (Sarmila et al. 2019). Torres-Toukoumidis et al. (2019) noted the importance of preparing a learning platform with gamification in the context of selecting for entrepreneurial competences. Isabelle (2020) further discussed the impact of gamification from an entrepreneurial education perspective, while also noting that supportive decision-making should also be analysed and evaluated. This discussion reveals that it would be good practice to launch an online gamification learning measure, as this is likely to have a supportive technological impact on entrepreneurial education and enable the development of actionable implications for pedagogical stakeholders and researchers.

Specifically, in the context of entrepreneurship education, for students and graduates, life has never been more uncertain: they need to be entrepreneurial in order to overcome new challenges, embrace constant changes and thrive in work and personal life. Therefore, as Zhang and Price (2020) proposed that pedagogical alignment is key to entrepreneurship education, and gamification has been acknowledged for its potential in enhancing learners’ performance and nurture their entrepreneurial competence with well-balanced alignment with other teaching modules (e.g. Aries et al. 2020; Isabelle 2020; Kariv et al. 2019).

## Methodology

### Research and data collection method

We adopted a mixed method in the present study, namely an open-ended text survey conducted with 91 students who participated in the game-based classroom, followed by in-depth interviews with 23 students who had completed the survey. This method allowed the authors to discern the hidden thoughts, emotions and behaviours of the respondents, and further enabled students to express their observations and opinions regarding the learning process via anonymised data collection, revealing key concepts and perspectives related to the entrepreneurship game. Based on the text mining outcome of the survey data, 23 participants were asked to complete a semi-structured questionnaire, which collected the students’ freely expressed intentions and opinions in order to analyse their views in depth. The perception-related data and information was contextual and situated, and consequently required the authors to guarantee that related circumstances would be brought into focus in order to produce the situated knowledge (Edwards and Holland 2013).

As the goal of the present research is to discern and reveal the students’ perceptions of the game-based learning module, which was done in order to evaluate how well the students could learn and achieve the intended learning outcome, all the students who studied this unit were asked to participate in the survey.

### Thematic analysis: Step 1

The open-ended text data of the survey was summarised for thematic analysis purposes. A coding process was utilised to enable the authors to identify patterns and themes within the interviewees’ statements (Auerbach and Silverstein 2003). Text mining software (NVivo 11) was used to develop the codes and to examine the patterns and relationships among the words, which further enabled the authors to analyse the participants’ profound perceptions, hidden thoughts and ideas. A list of frequently used word groups provided the basis for the interview guide used in Step 2.

### Thematic analysis: Step 2

A total of 23 students contributed to the in-depth interviews, which were conducted based on the interview guide developed from Step 1. The interviews were recorded, transcribed and coded. The keywords and links identified among the codes were then critically analysed with reference to the existing literature. Following this process, the findings and key themes that emerged were summarised to create a conceptual framework, which should be practically useful for designing a curriculum that enhances students’ active engagement and secure learning outcomes.

## Analysis

### Game design

One example of a type of game relevant to this field is a ‘game-type educational software that can analyse the behaviour of operators precisely.’ This prototype is designed as a business game played online. Multiple participants can play the game at the same time, which enhances both its instructive value and its learning outcomes.

Gee (2004: 86) discussed ‘a unique real-virtual story produces a new form of performance art co-produced by players and game-designers,’ and also he emphasised that ‘it is a form that has the potential to integrate pleasure, learning, reflection, and expanded living in ways that we expect from art.’ In line with this discussion, he suggested three key points for game design (Gee 2007: 24):

1. We learn to experience (see, feel, and operate on) the world in new ways.
2. Since semiotic domains usually are shared by groups of people who carry them on as distinctive social practices, we gain the potential to join this social group, to become affiliated with such kinds of people.
3. We gain resources that prepare us for future learning anti problem solving in the domain and in related domains.

More specifically, Prensky (2006: 104–105) introduced case studies on the positive impact of enjoyable computer game for young learners, who independently learned ‘the concepts of supply chains, division of labour, value added, supply and demand, business structure, control, full employment, vertical integration, wealth building, capital acquisition, dealing with corruption, making difficult decisions, ethical behaviour, god communication’.

The software created by the author team was based on the need to provide more actionable knowledge with critical importance to the subject of interest in an enjoyable atmosphere, which is capable of responding to the problems and issues depicted in a given scenario. The process of the participants’ activities is recorded and analysed with the game outcomes. We also paid attention to the learning environment, which enabled the students to enjoy the game as well as fully understand the relevant themes based on critical thinking in the context of a business scenario.

Relevant views regarding business include whether a person wants to engage in production to make products or wants to engage in sales to sell products are discussed and reflected on the game scenario for this pilot study (Prensky and Thiagarajan 2007); in making business scenario more realistic with stimuli for the participants, tolerance for risk refers to whether a person is capable of being adventurous or is instead more cautious by nature has been included in the game scenario.

### Planning

At the beginning of each class, we explained the content of the game and the intention behind the game design. We invited students to participate and asked for permission to collect data. The game consists of two stages. In the first stage, each player is given 30 in-game days to prepare for the opening of their own restaurant, which is a business activity and also the target of this game. When preparations for opening the restaurant have been completed, the players commence the second phase, which is the competition stage. This competition stage involves ‘in-turn’ activities lasting for three in-game months. During their turns, the players compete to attract and keep loyal customers. Throughout this process, the participants learn a holistic view of the 3Cs approach.

### Data collection

The players were asked what kind of business they wanted to run and then to conduct an overall evaluation of the game in the context of the online learning outcome. The survey consisted of two steps. Firstly, data was collected throughout their participation, including the progress of their gaming logs. Five of the 18 preliminary survey questions were randomly selected and displayed on the screen for each student. The second survey was conducted as a process of unit evaluation at the end of the module. During this evaluation, all students were asked to present their views and opinions, particularly as regarded their learning motivation, interactive online learning, and the ways in which gamification supported their understanding of the relevant theories, models, and business behaviour. These surveys were subsequently followed by in-depth interviews with 23 students who were randomly selected from the participants.

#### Game log and initial activities

Even though students seemed to enjoy the game, enjoyment of the online game was not the main focus of this activity. Instead, the diagnostic function is the core part of the game. The diagnosis of the situation is not displayed until the game is completed. To vary the participants’ segmentation and enhance the variety of the game, several questions were prepared to ensure that the players could play the game in different scenarios and arrive at different outcomes.

**Fig. 1:**
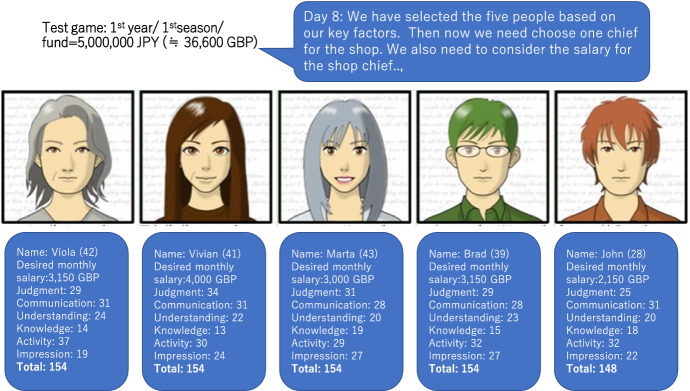
The five candidates appear based on the results chosen by the player

Throughout the game, the students were also provided with an opportunity to state their future vision and intentions for the company. Participants were further encouraged to analyse and discuss the economic situation of the restaurant market, along with various business-related elements. The selection of resources and materials is another key element of the game. There are a variety of resources that are necessary for opening the store, the first being people (human capital) with different titles and responsibilities. In total, 52 items were prepared and 20 of these items were displayed. The results differ depending on which of the following elements the player considers most important for the store manager. The game players chose store managers by considering the candidates’ ability and expertise. Figure 1 presents images of store manager candidates, whose profiles represented the basic information used by the players to decide on whom they would recruit for their businesses.

The five candidates appear in the game based on the chosen results. The person with the abilities closest to the answers provided in the earlier questions is given the job. As this is relevant to how the game operates in the next stage, the player needs to make this hiring decision carefully. The outward appearance, age and names of the candidates were set randomly; however, older candidates had higher abilities and salary on average. The question of which candidate was hired was also relevant to the diagnosis: if a person with a high salary and a high level of ability was chosen, this would increase monthly expenses, but could also increase sales.

The procurement of funds was also important in the preparations for opening the store. It was up to the players whether to choose equity investment or a loan; if the player chose equity investment, it would be necessary to distribute dividends.

#### Interim survey results

**Fig. 2:**
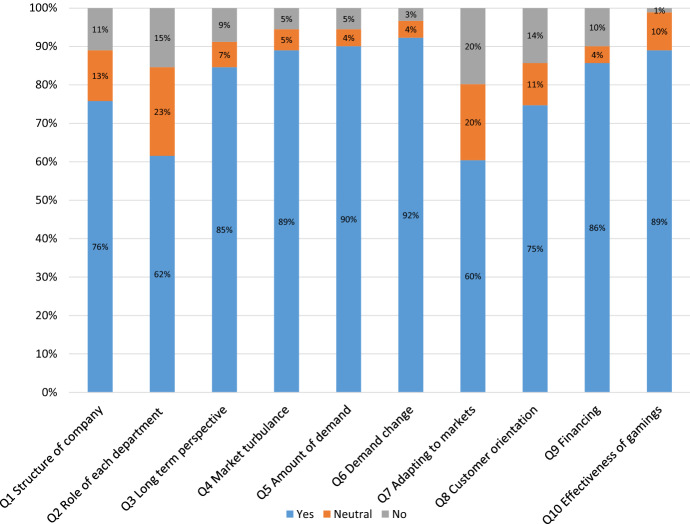
Students’ perspectives on effectiveness of learning outcomes for ten topics

The questionnaire was distributed to the 91 participants in the class and a mini-survey was conducted to reveal their basic thoughts and opinions on game-based active learning. The results are presented in Fig. 2. In the questionnaire, the questions were asked on the students’ perspectives how they evaluated the effectiveness of online game practices on ten learning topics in the entrepreneurial learning.

As can be seen from Fig. 2, almost all participants appreciated the game-based learning process (Mean of Q10 = 4.76) and evaluated it positively. In particular, they appreciated learning about market structure (Q4), demand (Q5&6), and the overall effectiveness and usefulness of games to deepen their understanding of business studies. Table 1 demonstrates two factors generated from their perceptions, which are (1) customer-oriented themes and (2) market-oriented themes, and relatively, the students highly appreciate the learning outcome on the customer-oriented themes (Mean = 4.52–4.78).

**Table 1:** Factors generated from the result of interim survey and descriptive statistics

Rotated component matrix^a^						
	Component					
	Factor 1	Factor 2	Min	Max	Mean	SD
Q5 Demanded amount from markets	0.925	0.338	1	5	4.69	0.985
Q6 Demand change in markets	0.915	0.229	1	5	4.78	0.814
Q4 Market turbulence	0.903	0.389	1	5	4.67	1.001
Q9 Financing	0.763	0.589	1	5	4.52	1.242
Q3 Long-term perspective	0.755	0.604	1	5	4.52	1.205
Q7 Adapting to markets	0.235	0.939	1	5	3.81	1.605
Q2 Role of each department in the company	0.321	0.916	1	5	3.92	1.500
Q8 Customer orientation	0.459	0.853	1	5	4.21	1.457
Q1 Structure of company	0.557	0.788	1	5	4.30	1.346
Q10 Effectiveness of gamings to enhance learning outcome			1	5	4.76	0.720

Extraction method: principal component analysis. Rotation method: varimax with Kaiser normalisation

^a^Rotation converged in three iterations

### Thematic analysis of open-ended survey

#### Step 1: overview of word cloud of open-ended text data

To provide an overview of the data set, an NVIVO word cloud was created to identify the key words used by the participants, while the groups of most frequently mentioned words are categorised and presented. From this process, it was found that the participants mostly perceived the impact of ‘changes’ on ‘event(s)’ and they appreciate the different scenarios got available depending on their decisions which helped the students to learn real business activities on the basis of their textbook-based knowledge. From this phase, key topics for in-depth interviews were developed, Collaboration with group mates with diversified values, Negotiation process, and Various events in different scenario settings. In addition to these three key themes, the interviewees were also asked about their free opinions on future potential of gamification in entrepreneurial learning.

#### Step 2: interview data analysis

The interview data is used to analyse students’ views and evaluations of the game-based learning module. Cai et al. (2016) emphasised that while the use of simulation and serious games for education has been popular, this has mainly taken place in language-learning classes and at the primary and secondary school levels (see e.g. Ibrahim 2019; Blume 2020; Peterson et al. 2020). This is despite the fact that López and Cáceres (2010) suggested a decade ago that the use of virtual games in social science education has to potential to contribute to students’ learning outcomes. Rippa and Secundo (2019) further noted that digital games can be part of a supportive toolkit to enhance entrepreneurship learning at universities.

In the following sections, we will reveal and develop an understanding of students’ views towards games in entrepreneurship classes based on revealed three key themes as discussed in the previous section, which was followed by further suggestions and recommendations from the participants for HE institutions when they attempt to expand the possibilities of implementing games in entrepreneurial classes.

#### Collaboration with group mates with diversified values

Students mentioned it was helpful to have diversity in the group, which enhanced their learning outcomes:

The discussion process is also a critical part of participating in games, I found it more helpful to enhance our learning outcome (Student 6).

In the international classroom, it was fun to collaborate with classmates who had different values and backgrounds (Student 11).

As Dell’Aquila et al. (2019) discussed, participating in digital gaming environments can also provide opportunities for the teams to engage in interethnic conflict management. Moreover, enhancing the learning outcomes based on intelligent systems and advanced technology can contribute to a deeper understanding of entrepreneurial learning when based on activities involving diverse team members; for instance, Student 24 made a comment *‘*It was exciting for us to collaboratively work with other classmates with different opinions and views, even with new students, it was easier for me to start communication on the game platform’.

#### Negotiation process and critical thinking

Students acknowledged the importance of the negotiation among members with different levels of ‘risk tolerance’ in the learning process of the entrepreneurial learning module:

‘Before participating in this game, I did not understand the meaning of “risk tolerance”’ (Student 3).

‘I knew the term “risk taker” from textbooks, but it was interesting to understand it from the communication among the team members while we made decisions about our choices’ (student 16).

Sanina et al. (2020) further suggested that digital games can promote a co-creative learning approach in the classroom, which is contributed to by both students and tutors. This concept is supported by some students:

‘I learned the meaning of critical thinking from the game!’ (Student 10).

‘I understand what critical thinking means. Through gaming activities, I learned how to forecast, how to coordinate, how to find the best options based on the given conditions and information….’ (Student 23).

Moreover, participating in gaming has enhanced the students’ critical thinking which is also supported by scholars who evaluated the learning modules based on interactive gaming and learning materials (e.g. Huang and Yeh 2017; Isabelle 2020; Rosli et al. 2019).

#### Online games in remote learning with entrepreneurship scenarios

It has been noted that gamification approaches should ensure that the learning process involves imprinting textbook-based theories and models via engaging in real activities as gamers. As Tang and Taguchi (2020) discussed, designing a scenario-based digital game has potential in various fields of study; this was widely and favourably acknowledged by the participants. Schulz et al. (2019) agreed that scenario-based serious games had further potential to enhance learners’ performance in entrepreneurship education.

Ratten and Jones (2020) also discussed entrepreneurship and management education could enhance the learning effectiveness by using interactive game with different scenarios especially during the remote teaching.

‘It was exciting to experience a series of different scenario games in the class’ (Student 17).

‘From a learner’s point of view, experiencing different scenarios for the games—and also I felt I was controlling the whole game process as an owner of the game, which encouraged my motivation to learn’ (Student 3).

‘It was good for us to experience a variety of scenarios to which we needed to respond based on our critical discussions and decisions, I really felt as if I had a responsibility as a shop owner’ (Student 18).

As Ratten and Jones (2020) discussed, recent technologies have enabled business education by conducting remotely, and the impact of Covid-19 on entrepreneurship education needs to be explored and discussed preparing for the phase of the ‘new normal’ to develop actionable implications for the ICT-based education such as online gaming. Some students made the relevant comments on the theme:

‘…Online game during remote session worked well, we do not worry about physical distancing, and we can enjoy realistic scenarios during the games.’ (Student 23).

‘I actually participated in a gaming during the session last term in the classroom when it was before lockdown, but I felt I was engaged more this semester, perhaps gaming is a good stimulus for us to study positively!’ (Student 14).

However, some other students made comments in a clearer way implying that logical connections between online game and COVID-19 lockdown:

‘….Simply, we need interactions. ….purely I enjoyed online game during remote learning. We are required to stay home and are limited social interaction, virtual interaction supported my mental health!!’ (Student 19).

‘ Online game with team collaboration was meaningful for me….not only because it was a good learning tool, but also because interactive learning with a touch of real business scenario made me excited during the class’ (Student 21).

As Oe et al. (2020) suggested, most of pedagogists in entrepreneurship education perceive gamification as useful in reinforcing key themes and topics after having learnt them through traditional means, at the same time, they emphasised how to design an effective gamification-based curriculum during remote teaching is still on our urgent agenda. The comments (Students 19 and 21) imply that the learners’ perceptions of virtual interaction with positive impact during COVID lockdown also should be considered in designing an online game-based module during the remote learning phase.

#### Agenda for future application of gaming in remote learning

At the end of the in-depth interviews, the interviewers allowed some free discussion time to collect comments on the future potential of gaming in the remote learning environment, with a specific focus on the impact of COVID-19.

A classroom is not just a place but is instead a hub of interaction and a nurturing learning atmosphere based on collaborative communication among students and tutors (Alles et al. 2019). Who suggested that establishing a positive learning atmosphere and conversation culture during in-class activities is one of the critical factors to supporting students’ learning outcomes.

‘Learning is learning, but I prefer physical classrooms, where we can communicate closely and learn better and more!’ (Student 8).

‘In mid-March–April, our classes were all switched to remote learning, and it has been a bit difficult to keep my motivation to learn, even though we are familiar with using the internet, SNS etc.’ (Student 15).

‘Participating in games enabled us to play as a team with group members, which was good. Virtual learning classroom needs some more interactive activities to make us feel connected with classmates and teachers’ (Student 16).

Conradie et al. (2020) suggested online gaming has a potential as an educational tool in enhancing entrepreneurial skills, entrepreneurial pedagogy needs to catch up the most recent advanced technological innovation which also requires effort to review and redesign online game-based teaching modules. Student 20 made a comment implying learners’ high expectation on this saying *‘*I look forward to attend a class with further improved online games next semester!’.

#### Additional support: writing skills

Interestingly, as Reynolds and Kao (2019) discussed, the digital game-based learning module requires direct written feedback to communicate with the students. In addition to tutors providing feedback to the students, some interviewees stated that they felt additional writing activities would be helpful for them in imprinting their learning and experiences throughout their participation in the games.

‘While participating in games, sometimes I felt uncomfortable, as I did not have enough time to take notes what I was learning… It would be helpful for me to take notes, which should be the basis for reviewing and reflecting what I am learning’ (Student 9).

‘Some of my classmates created reviewing notes, which seemed to be useful for reviewing the learning process’ (Student 1).

‘Eventually, I found communicating using text was supportive for me to enhance my learning outcome. Also, I found that the writing activity itself was good for me to imprint the basic theories and knowledge in the context of the game scenario’ (Student 17).

This point is supported by the discussion presented by Nouri (2019), who emphasised the importance of enhancing multimodal literacy and critical thinking. While we have discussed the impact of gamification in higher education institutions, we have been inclined to overlook the skill of writing as a mediator to support the learning process; this has thus been considered one of the critical agenda topics when we aim to enhance the implementation of games in class activities.

Gaming first, theories next: requirement of the ‘cyclic approach’.

While the effectiveness of interactive multimedia-based learning approach has been discussed and evaluated in the higher education context, precisely which kind of teaching menu/package is required to enhance the positive outcomes of game-based learning has not thus far been researched. On this theme, quite a few students stated their acknowledgement that a ‘well-balanced-sandwich’ or ‘cyclic’ module was effective.

‘Doing the games themselves was fun, but it was helpful to continue classroom learning, games, and class discussion, the process worked well’ (Student 15).

‘Never-ending learning process, games can be stimuli for our learning motivation, cyclic learning modules were helpful for us’ (Student 4).

‘Especially, activities first, then learning theories and models worked very well to me!’ (Student 6).

Student 16 also stated: *‘*During social distancing because of COVID-19, participating in the games worked especially well, but perhaps if there were not also drop-in sessions and livestream seminars as virtual classrooms, the effectiveness of these games could have dropped hugely’ (Student 16)’.

In addition, some other students clearly stated the ‘gaming first, theories next approach’ worked well in enhancing their understanding the relevant knowledge and experiences:

‘Gaming suggested me to ‘use our brain more’ and we discussed well in the group. I felt it was a good opportunity to act strategically and critically.’ (Student 20).

‘Sometimes it was not easy for me to read a textbook first. But in this class, we participated games first, which was followed by theoretical modules….I liked this approach, as it was much easier for me to understand what the text book says…’ (Student 11).

## Discussion

**Fig. 3:**
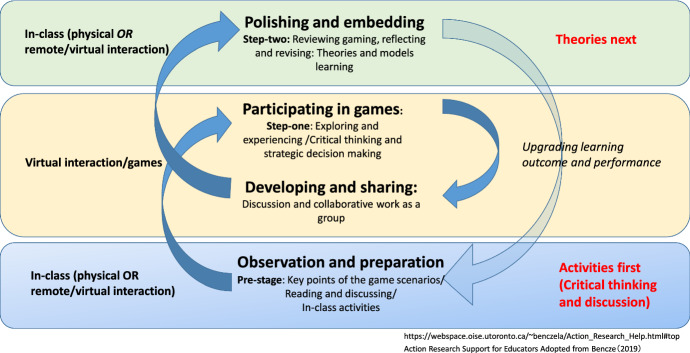
Action learning circuit with games (observation → participation → developing → polishing and embedding)

In this section, we summarise research findings and present a proposed conceptual model to enhance implications for online game-based pedagogy. Figure 3 demonstrates key themes and backbones for game design in supporting students’ learning journey with a cyclic approach in deepening students’ critical thinking and application of theories in entrepreneurship learning. Students’ comments can be basically categories in three phases: pre-gaming, during, and post-gaming steps.

And moreover, students suggested a holistic reviewing approach to revise by adding scenarios of entrepreneurial behaviour in the business context, which can engage students in learning activities based on a collaborative learning atmosphere with independent learning commitment (Table 2).

**Table 2:** Summary of research findings

Phase	Key theme	Comments
Pre-gaming phase	- Gaming first, theories next	Student 4, 6, 11, 15, 16, 20
	- Preparation of a variety of different scenarios	Student 3, 14, 17, 18, 19, 21, 23
	- Requirement of a ‘cyclic approach’ (review of the previous gaming and learning outcome of students)	Student 4, 6, 11, 15, 16
During game phase	- Collaboration with group mates with diversified values	Student 6,11,21,24
	- Negotiation process and critical thinking	Student 3, 10, 16, 20, 23
	- Usefulness of online games with different scenarios in remote learning/COVID-19 lockdown	Student 3, 14, 17, 18, 19, 21
Post-gaming phase	- Review and redesign of games. Future application of gaming in remote learning/cyclic approach to develop next versions based on the gaming experiences	Student 8, 15, 16, 20
	- Additional support	Student 1, 9, 17
	- Gaming first, theories next	Student 4, 6, 11, 15, 16, 20

Based on the above analysis, a conceptual framework for implementing games in the learning process was developed and is presented in Fig. 3.

Entrepreneurial education is conventionally based on the teaching of relevant theories and models, which students learn to critically discuss and evaluate before applying them to business cases. Participants agreed on the positive impact of gamification, which engaged them in business learning by enhancing their critical thinking practices and could form a later basis for the learning of entrepreneurial theories and models, and in doing so, as Fig. 3 demonstrates, the clear pathways following observation, participation, developing, and polishing steps will support students’ learning performance.

## Conclusions

### Contribution

Entrepreneurship and business education using online games will improve students’ literacy concerning finance and economy in the future. As Ponticorvo et al. (2019) emphasised, it is critical for educational game designers to consider not only the game contents and scenarios, but also the psychological impact on game participants.

Traditionally, business education primarily involves the mastering of theoretical economics and business administration knowledge; by contrast, gamification enables students to think critically based on certain ‘behaviours’, which involve ways of thinking regarding competitive business scenarios. This approach could represent a different approach to conventional business education.

From this study, several critical points have been developed. First, it has been found that the online game, along with its mutual interactive learning atmosphere, is a useful tool to enhance learning outcomes; this is particularly true given the ongoing impact of the COVID-19 situation, which requires higher education institutions to deliver classes remotely. The participants agreed that gamification had a positive impact in terms of engaging them in business learning and stated that critical thinking in the context of virtual gaming could form the basis for learning about management theories and models. At the same time, the participants also emphasised the importance of cyclic learning modules that incorporate both games and ‘physical’ interaction in order to embed the learning outcome in the form of solid knowledge and experiences.

Overall, the status of online game support in entrepreneurship and business studies are still in the experimental phase. As Prensky (2006), Prensky and Thiagarajan (2007), Gee (2007), and Gee and Hayes (2012) pointed out, the usefulness of serious games has been discussed, whereas it is hard to say that school teachers are now familiar with handling the educational games in the classrooms. In fact, participants are well engaged in online game training sessions as a part of building new curriculum; however, implementations of online gaming for entrepreneurial and business studies have not progressed sufficiently so far. Therefore, from the study outcome, we would provide some suggestions for each stakeholder, tutors, curriculum designers, and pedagogical policy makers.

Tutors who operate online games in classrooms should understand the game structures and also should be involved in game design sharing the experiences attained in the classrooms and students’ feedback.

For curriculum designers, it is essential for them to co-work closely with the operators of the games in classrooms and implement the online games embedded in the whole module delivery to support the students’ learning journey. It is also critical for them to check the participants’ responses to online games and students’ learning performance to review and improve the online game designs.

As noted, online game implementation is not that speeded up in higher education organisations, but on the other hand, for private companies, there have been various accumulations of online game applications that have realised in customised business games for different contexts and cases. So as a policy makers’ intervention, the relevant pedagogic stakeholders should share the advanced information and experiences from private sectors to support online game designing in the universities and other educational organisations. It can be said that it is in a transitional period for pioneering online game in entrepreneurship and business education, so some more collaborative consortia or initiative involving pedagogic stakeholders surrounding universities to enhance effectiveness of application of online games in the module would be required.

### Limitations

The research has developed a guideline of how to utilise online gaming for the entrepreneurship and business education at universities, and the outcome of the study should be a useful discussion tool for relevant researchers and pedagogists. However, we still acknowledge some limitations to consider.

Firstly, the number of data sets is small; therefore, it is necessary to verify the research outcome with some more data sets to develop robust implications and recommendations for the field of study. Secondly, the authors have not conducted detailed examinations based on the students’ proficiency level, technological preparedness, and demographic factors. Thirdly, this research was conducted only with the students who participated in the online gaming during the unit delivery. It would be critical to collect data from other pedagogic stakeholders, such as tutors, module designers, and policy makers who should be involved in the collaborative partnership to enhance usefulness of online game in entrepreneurship and business education.

As for the effect of ‘games first, theories next’ approach, this approach could be compatible with the researchers’ estimation that it could be supportive for students, especially those who have little experience in business management to be engaged in a virtual business experience in the first place. And the research outcome indicates that online exercise seems to be a good trigger to deepen their understanding of the relevant theories and models. But it is again necessary to evaluate the outcome of participating online gaming with some data to review the effectiveness of the approach proposed from this study.

### Further research opportunity

A theme of future importance for pedagogists will be that of how to enhance students’ learning outcome from online game-based modules. As Fig. 3 indicates, it is critical for the module designers to consider three phases: pre-gaming, during, and post-gaming stages. Also, it is inevitable to embed group collaboration as a basis for interactive learning especially during remote learning because of COVID-19. To enhance students’ engagement, another factor we should pay attention to is how to include different business scenarios based on live case studies as active learning materials. The activities, in real business settings, will yield fruitful results beyond academia by nurturing future businesspersons who are able to act strategically and proactively in a real business world.

Implementation of online game during teaching enables tutors to collect objective data from game logs which record each student’s business behaviour and decision-making process in responding to a game scenario. In subsequent studies, it has to develop an evaluation framework of the online game implementation. Factor analyses have been conducted to build a structural equation modelling for examining the effects of online games on students’ learning outcome; however, the degree of conformity has not been agreed on mainly because we have not had a set of variables to adopt and what path diagrams should be drawn. To support the students’ learning journey during lockdown in more depth, data analysis on behavioural bias should also be conducted to develop actionable implications with new game scenarios in further research opportunities. And from there, we believe that we can elucidate the mechanism of human behaviour change in business contexts, which should be the basis for entrepreneurial education.
